# Transcriptomic and Ultrastructural Analyses Reveal the Mechanisms of Accelerated Depuration Induced by *Phyllodium pulchellum* Extract in the Freshwater Snail *Bellamya purificata*

**DOI:** 10.3390/ani16101490

**Published:** 2026-05-13

**Authors:** Zhiqiang Wang, Enjie Chua, Fuguang Luo, Xiaoyun Zhou, Jie Huang, Jinxia Peng, Xianhui Pan, Yanhong Wen

**Affiliations:** 1 Liuzhou Aquaculture Technology Extending Station, Liuzhou 545006, China; wangzhiqiang2019@foxmail.com (Z.W.); luofuguang3563@163.com (F.L.); huangjie_2026@foxmail.com (J.H.); 2 Guangxi Key Laboratory of Marine Environmental Disaster Processes and Ecological Protection Technology, College of Marine Sciences, Beibu Gulf University, Qinzhou 535011, China; chua.jie.6@gmail.com; 3 College of Fisheries, Huazhong Agricultural University, Wuhan 430070, China; zhouxy@mail.hzau.edu.cn; 4 Guangxi Key Laboratory of Aquaculture Genetics and Breeding, Guangxi Academy of Fishery Sciences, Nanning 530021, China; pengjinxia@gmail.com (J.P.); panxhfisher@126.com (X.P.)

**Keywords:** *Bellamya purificata*, depuration, *Phyllodium pulchellum*, transcriptomics, genotoxic stress

## Abstract

The commercial viability of *B. purificata* relies on efficient depuration. While *P. pulchellum* extract dramatically accelerates this process, its physiological and molecular mechanisms remain unknown. To address this, we integrated behavioural, histological, ultrastructural, and transcriptomic (RNA-seq) analyses to characterize the acute stress and subsequent recovery phases of *B. purificata* exposed to the botanical extract. Extract exposure induced severe neuromuscular hyperextension response, with histological analysis revealing acute interstitial oedema and neuronal chromatolysis. This structural damage caused sustained cephalopodium extension and muscle fibre uncoupling. Transcriptomic profiling linked this neuromuscular dysfunction to conserved calcium-dependent adrenergic signalling modules and profound endogenous neuroendocrine disruption. The extract also induced cellular stress, downregulating the apoptosis inhibitor *BIRC7* and eliciting transcriptomic signatures consistent with a DNA damage response. Crucially, during the short-term recovery phase, surviving tissues mounted a robust transcriptomic repair response. The snails systemically suppressed the cell cycle via *MCM2-6* downregulation while upregulating *GADD45* and *RAD54B*, suggesting a prioritization of genomic repair alongside partial morphological reorganization. Ultimately, *P. pulchellum* accelerates depuration via acute phytochemical stress and neuromuscular dysfunction. Importantly, this stress is accompanied by a highly coordinated transcriptomic repair response and partial short-term restoration, providing foundational molecular insights essential for evaluating and optimizing botanical depuration protocols.

## Simple Summary

The freshwater snail *Bellamya purificata* is a key ingredient in the popular Luosifen food industry, but the necessary gut-clearing process, called depuration, is traditionally slow, inefficient, and often leads to high snail mortality. To solve this industrial problem, we tested an extract from the medicinal plant *Phyllodium pulchellum* to accelerate waste expulsion. Our study, which integrated advanced RNA sequencing and microscopy, found that the extract works by rapidly over-stimulating the snail’s nervous system, causing neuromuscular hyperexcitation through a serotonergic-driven mechanism. This stimulation forces the snails into a sustained, hyper-active state, allowing for rapid and complete gut clearance. Although the extract induces transient structural stress, the cells activate a robust DNA repair program (upregulating *GADD45* and *RAD54B*) in the recovery phase, demonstrating the process is transient and reversible. These findings provide a scientific foundation for establishing a safe, highly efficient processing protocol for the commercial snail industry.

## 1. Introduction

In China, the local term ‘Luoshi’ collectively refers to a group of freshwater snails belonging primarily to the genera *Bellamya* and *Cipangopaludina* (Family: Viviparidae; Class: Gastropoda; Phylum: Mollusca). This diverse group encompasses several economically significant species, including *Cipangopaludina cathayensis*, *Cipangopaludina chinensis*, *B. quadrata*, *B. aeruginosa*, and *B. purificata* [1]. As widely distributed benthic invertebrates, these snails are important components of aquatic ecosystems and represent a valuable agricultural resource [1]. In recent years, *B. purificata* has become particularly sought after as the core raw material for Liuzhou Luosifen (snail rice noodles), a regional specialty industry that generated over 75.9 billion RMB in full-chain sales revenue in 2024 [2,3].

Because of their benthic feeding habits, the snails accumulate mud, algae, and detritus within their digestive tracts, which imparts an undesirable earthy and muddy flavour to the food products [4,5]. Consequently, a critical pre-processing step known as depuration (commonly referred to in the industry as ‘purging’ or mud expulsion) is required to empty the intestinal contents and ensure optimal flavour and safety. Conventional depuration methods rely on holding the snails in clean water for 2 to 3 days. However, this extended duration, coupled with the snails’ slow metabolic rate [6], often leads to hypoxia, necessitating frequent water changes. Prolonged holding times also inevitably result in high mortality rates, and the subsequent spoilage severely compromises the quality of the final product [7].

To address this industrial bottleneck, recent practical applications have identified *Phyllodium pulchellum* (syn. *Desmodium pulchellum*), a medicinal legume traditionally used in Guangxi to treat liver ailments [8,9], as a highly effective depuration stimulant. Exposure to aqueous extracts of *P. pulchellum* rapidly induces an intense behavioural response state in *B. purificata*, characterized by rapid shell emergence, sustained body extension, and continuous mud expulsion [10]. This botanical intervention drastically reduces the required depuration time at remarkably low concentrations, significantly improving processing efficiency and allowing for the swift identification and removal of dead or empty shells [10].

Despite these clear industrial benefits, the physiological and molecular mechanisms driving this plant-induced behavioural shift remain poorly understood. Such profound behavioural modifications are likely mediated by the molluscan neuroendocrine system, which synthesize a wide array of neurotransmitters and hormones to regulate physiological activities. For instance, common neurotransmitters identified in bivalves include classic neurotransmitters (e.g., ACh, CAs, and GABA) and various neuropeptides [11,12,13,14], alongside broader systemic hormones [15,16,17]. Detailing this baseline neuroendocrine complexity is crucial, as these specific signalling molecules fundamentally govern the neuromuscular contractility and systemic stress adaptation that are directly disrupted during botanical-induced depuration. Furthermore, as *P. pulchellum* is rich in bioactive phytochemicals like alkaloids and flavonoids [18,19], its potent stimulatory effect at acute doses likely triggers complex systemic stress responses, potentially involving intracellular ion dysregulation and subsequent cellular damage. High-throughput RNA sequencing (RNA-seq) has proven to be a highly sensitive and accurate tool for characterizing these complex, multi-pathway stress responses in non-model organisms [20,21,22].

Therefore, this study aims to elucidate the multi-layered response of *B. purificata* to *P. pulchellum* extract. By integrating histological examination, transmission electron microscopy (TEM), and transcriptomic profiling, we characterized the morphological and molecular shifts during the acute exposure (Treated) and subsequent Recovery phases. Specifically, we sought to infer the transcriptomic pathways associated with the observed neuromuscular locking, evaluate the extent of phytochemical-induced cellular stress, and uncover the delayed genomic repair mechanisms initiated upon the extract’s removal. These findings will provide a comprehensive molecular foundation for optimizing this processing technology, thereby supporting the high-quality development of the snail aquaculture and food processing industries.

## 2. Materials and Methods

### 2.1. Preparation and Chemical Profiling of P. pulchellum Extract

Whole plants of *P. pulchellum* were naturally dried and mechanically crushed into a fine powder (30–80 mesh). The aqueous extract was prepared by adding 20 g of the plant powder to 1 L of distilled water, which was subsequently boiled for 15 min. The resulting decoction was filtered to remove particulate residue and allowed to cool to room temperature prior to use in the exposure experiments. For the identification of major bioactive constituents, the prepared *P. pulchellum* extract was subjected to ultra-high-performance liquid chromatography–tandem mass spectrometry (UHPLC-MS/MS) conducted by the Beijing QingXi Technology Research Institute (Beijing, China) using a Q Exactive Plus Orbitrap high-resolution mass spectrometer (Thermo Fisher Scientific, Waltham, MA, USA). Data processing via Compound Discoverer 3.2 identified the primary constituents, which were dominated by flavonoid glycosides and quaternary ammonium alkaloids, including rutin and betaine.

### 2.2. Snail Collection and Experimental Setup

Healthy *B. purificata* (average wet weight: 4–5 g) were harvested from aquaculture cages at the Snail Aquaculture Demonstration Base of Liuzhou Guzhiyun Agricultural Development Co., Ltd. (Liuzhou, China). To accurately replicate the standard industrial post-harvest supply chain, the exterior shells of all snails were mechanically brushed clean immediately upon harvest before being transported directly to the laboratory. To preserve their natural gut contents and baseline physiological state for the depuration assay, no extended laboratory acclimation or fasting period was performed. Upon arrival, the snails were randomly allocated into 5 L plastic containers at a stocking density of 15 snails per container, each containing 2.5 L of fresh water. A total of 9 containers were utilized to provide three independent replicate containers for each of the three experimental groups. During the experimental procedures, the snails were maintained under an ambient laboratory photoperiod. The water was continuously aerated to maintain dissolved oxygen saturation, and the temperature was maintained at 21.5 °C.

### 2.3. Extract Exposure and Tissue Sampling

To stimulate the acute stress response, the prepared *P. pulchellum* extract was added to the 5 L holding containers, yielding a standardized final exposure concentration of 80 mg of dry plant material per litre of water (0.08 g dry plant/L). During the exposure period, macroscopic behavioural and physiological responses, including righting reflex, resting posture, and mucus secretion, were continuously monitored and recorded at 10-min intervals.

The experimental design consisted of three distinct sampling groups to capture the acute stress and recovery phases. The Control group (0 h) consisted of snails sampled immediately prior to the addition of the extract. Based on the behavioural observations establishing 40 min as the peak of the acute depuration response, snails in the Treated group were sampled after 40 min of continuous exposure to the extract. Finally, for the Recovery group (2 h), snails were exposed to the extract for 40 min, subsequently transferred to clean, aerated water, and allowed to recover for 2 h prior to sampling. To ensure robust statistical power and account for potential container effects, biological replicates (n = 3) were defined by the three independent replicate containers per group. From each container, cephalopodium tissue was dissected on ice from three randomly selected snails and pooled into a single sample tube, meaning a total of 9 snails were sampled for downstream molecular and histological analysis per experimental group.

### 2.4. Histological and Ultrastructural (Tem) Preparation

For light microscopy, cephalopodium tissues (n = 3 per group) were immediately fixed in 4% paraformaldehyde. The samples were later dehydrated through a graded ethanol series, embedded in paraffin, sectioned, and stained with Haematoxylin and Eosin (H&E) using standard manual histological protocols to assess general tissue architecture. To specifically evaluate neuronal integrity, adjacent sections were subjected to Nissl staining. Briefly, sections were stained with 1% cresyl violet solution, rinsed in distilled water, and differentiated in 95% ethanol until the background was visually clear. Slides were then dehydrated, cleared in xylene, mounted with a resinous medium, and evaluated under a bright-field light microscope. The extent of tissue damage, including chromatolysis and interstitial oedema, was systematically assessed across multiple fields of view.

For Transmission Electron Microscopy (TEM), distinct tissue sections from the cephalopodium muscle (n = 3 per group) were diced into small blocks (approximately 2 mm × 2 mm × 1 mm) and immediately submerged in 2.5% glutaraldehyde for primary fixation at 4 °C overnight. The samples were then washed in 0.1 M phosphate buffer (pH 7.4) and post-fixed with 1% osmium tetroxide for 2 h. Subsequently, the tissues were dehydrated through a graded ethanol series, transitioned through pure acetone, and embedded in epoxy resin. Ultrathin sections (60–80 nm) were obtained using an ultramicrotome, double-stained with 2% uranyl acetate and lead citrate, and examined using a Hitachi transmission electron microscope (Hitachi, Tokyo, Japan) operating at an accelerating voltage of 80.0 kV.

### 2.5. Total RNA Extraction and Quality Control

Transcriptomic analysis was conducted in collaboration with Wuhan Maiwei Metabolic Biotechnology Co., Ltd. (Wuhan, China). Total RNA was extracted from the snap-frozen, pooled head-foot tissues using TRIzol reagent (TaKaRa Bio Inc., Dalian, China) following the manufacturer’s instructions. RNA quantity and purity were assessed using a NanoDrop 2000 spectrophotometer (Thermo Fisher Scientific), ensuring an OD260/280 ratio between 1.9 and 2.0. RNA integrity was verified via 1% agarose gel electrophoresis prior to downstream applications.

### 2.6. cDNA Synthesis and High-Throughput Sequencing

Genomic DNA was eliminated, and first-strand complementary DNA (cDNA) was synthesized using a PrimeScript RT reagent kit (TaKaRa Bio Inc.) utilizing 1 µg of high-quality total RNA per reaction. The thermal cycling protocol consisted of 37 °C for 15 min, followed by enzyme inactivation at 85 °C for 5 s. Subsequently, sequencing libraries were constructed and subjected to high-throughput sequencing in triplicate on a DNBSEQ-T7 platform (MGI Tech Co., Ltd., Shenzhen, China) utilizing reagents and services from Frasergen Biotechnology (Wuhan, China). The resulting raw sequencing reads are available in the NCBI database under BioProject accession number PRJNA1442220.

### 2.7. Data Processing and Sequence Annotation

Raw sequencing reads were initially evaluated for quality using FastQC v0.11.5. Adapter sequences and low-quality bases were trimmed using the NGS QC Toolkit to obtain high-quality clean reads. The clean reads were subsequently mapped to the *B. purificata* reference genome (CNGBdb accession: CNA0142815) using HISAT2 v2.1.0. For comprehensive functional annotation, sequence alignments were performed against multiple public databases, including the NCBI Non-Redundant Protein Database (NR), NCBI Nucleotide Database (NT), Swiss-Prot, Pfam, Clusters of Orthologous Groups (COG/KOG), Gene Ontology (GO), and the Kyoto Encyclopedia of Genes and Genomes (KEGG). BLASTX v2.13.0 and Diamond v2.1.8 algorithms were employed with strict E-value thresholds (10^−5^ for general BLAST searches and <1 × 10^−10^ for NR, KEGG, Swiss-Prot, and COG). Pfam annotations and GO classifications were conducted using HMMER v.3.3.2 and WEGO v2.0, respectively.

### 2.8. Differential Expression and Enrichment Analysis

Gene expression levels were estimated by calculating the Fragments Per Kilobase of transcript per Million mapped reads (FPKM). Differential expression analysis between the experimental groups (Treated vs. Control; Recovery vs. Treated; Recovery vs. Control) was performed using the DESeq2 v1.42.0. Genes meeting the criteria of *p* < 0.05 and |log_2_FC| > 1 were defined as differentially expressed genes (DEGs). To ascertain the biological functions and pathways driving the physiological responses, GO enrichment analysis of the DEGs was performed using GOseq v1.54.0, utilizing Fisher’s exact test (*p* ≤ 0.05) to identify significantly enriched terms. Pathway enrichment analysis was conducted using KOBAS 2.0 to identify significantly enriched metabolic and signal transduction pathways against the KEGG database, with statistical significance established at a False Discovery Rate (FDR) ≤ 0.05.

### 2.9. Validation of Transcriptomic Data via RT-qPCR

To validate the accuracy of the RNA-seq data, seven key differentially expressed genes (DEGs) were selected for quantitative real-time PCR (RT-qPCR) analysis. Because several of these seven selected genes exhibited significant differential expression across multiple experimental comparisons (i.e., Treatment vs. Control, Recovery vs. Treatment, Recovery vs. Control), this validation yielded a total of 10 transcriptomic comparison points for the final correlation analysis. Total RNA was extracted using the EASYspin tissue/cell RNA rapid extraction kit (RA105-01, Beijing Aidlab Biotechnologies Co., Ltd., Beijing, China). First-strand cDNA was synthesized using the One Step First-Strand Synthesis MasterMix (MT201, Hece Biotechnology Co., Ltd., Beijing, China). Real-time quantitative PCR was conducted on an ABI-Q5 real-time PCR system (Applied Biosystems, Foster City, CA, USA) using the 2× T5 Fast qPCR Mix (SYBR Green I) (MT301, Hece Biotechnology Co., Ltd., Beijing, China) in a 20 µL reaction volume. The thermal cycling parameters included an initial denaturation at 95 °C for 1 min, followed by 40 cycles of 95 °C for 15 s, 60 °C for 15 s, and 72 °C for 30 s. A continuous melt curve analysis (50 °C to 95 °C) was performed post-amplification to confirm primer specificity. The *β-actin* gene was employed as the internal reference gene for normalization. Relative gene expression levels were calculated utilizing the 2^−ΔΔCt^ method, assuming an amplification efficiency of 100%. All primer sequences used for RT-qPCR validation are provided in Supplementary Table S1.

## 3. Results

### 3.1. UHPLC-MS/MS Characterization of the P. pulchellum Extract

To determine the principal bioactive constituents of the *P. pulchellum* extract, UHPLC-MS/MS was conducted. The phytochemical profile revealed a complex mixture dominated primarily by flavonoids and alkaloids. Based on relative abundance, the most prominent compound identified was the flavonoid glycoside rutin (23.65%), followed by the alkaloid betaine (14.25%). Other major constituents included kaempferol-3-O-rutinoside (12.29%), kaempferol 3-glucorhamnoside (5.76%), morin (5.24%), quercetin (3.29%), and hyperoside (2.71%). The full chromatogram and comprehensive list of identified compounds are provided in Supplementary Figure S1 and Supplementary Table S2, respectively.

### 3.2. Behavioural Response of B. purificata

**Figure 1:**
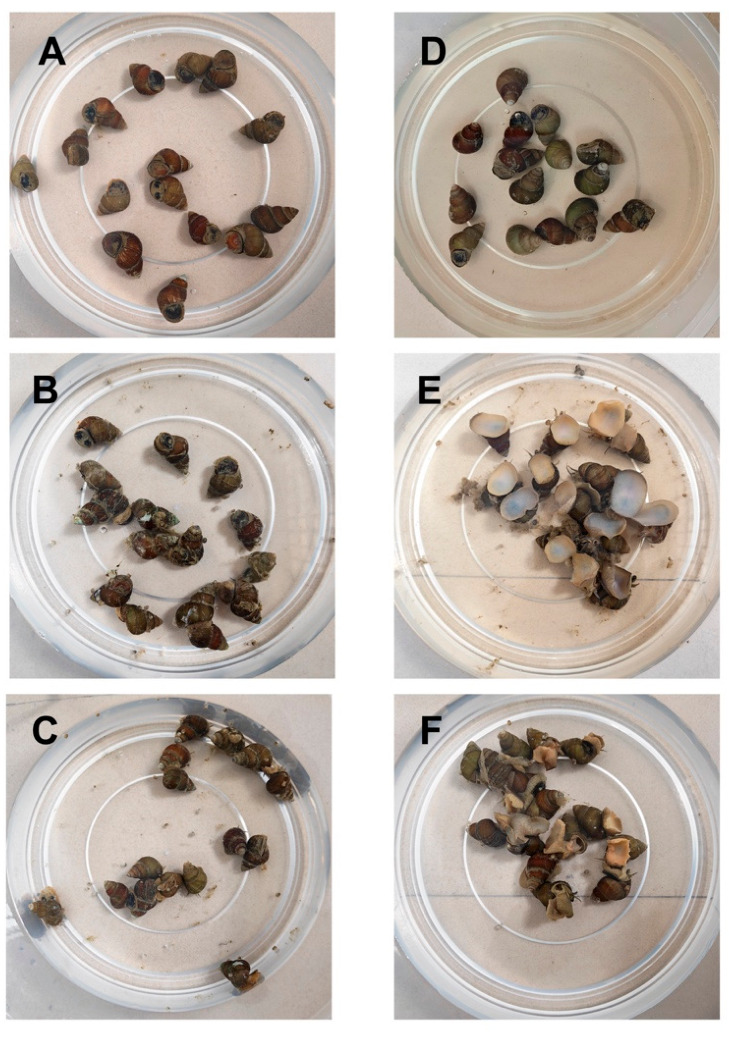
Behavioural and morphological responses of *B. purificata* following exposure to *P. pulchellum* extract. **Left** Column (Control group): (**A**) 0 h showing normal, unattached resting posture; (**B**) 40 min maintaining normal positioning with minimal basal excretion; (**C**) 2 h 40 min demonstrating full environmental adaptation with active substrate attachment and locomotion. **Right** Column (Treated group): (**D**) 0 h showing normal resting posture prior to treatment; (**E**) 40 min exposure exhibiting acute neuromuscular locking, complete cephalopodium extension, and massive accumulation of depurated matter; (**F**) 2 h recovery showing cessation of active depuration and partial cephalopodium retraction without full active attachment.

Initially, snails in both the Control and Treated groups exhibited a normal resting posture, remaining sensitive to the novel environment without immediate firm attachment to the substrate (Figure 1A,C). Behavioural observations were continuously recorded at 10-min intervals. By 40 min, the Control group had fully acclimated, maintaining normal opercular positioning with minimal activity (Figure 1B). In stark contrast, snails exposed to the *P. pulchellum* extract exhibited a progressive behavioural shift that peaked at the 40-min mark. At this peak, the treated individuals experienced a complete loss of the righting reflex (reaching a 100% loss rate) and were predominantly observed in a rigid, inverted position with the cephalopodium fully extended (Figure 1E). Concurrently, excessive stress-induced mucus secretion and an accumulation of initial basal excretion were observed in the treatment water. This physical output vastly exceeded the minimal basal excretion seen in the Control group, occurring as a direct mechanical consequence of the severe neuromuscular locking rather than a coordinated physiological depuration response. Because this 40-min timeframe represented the absolute peak of the acute physiological stress response, it was established as the precise endpoint for tissue sampling. Following the transfer to fresh water and a subsequent 2-h observation period, the Control group displayed normal active locomotion and firm attachment to the substrate (Figure 1C). Conversely, all the individuals in the Recovery group (Figure 1F) ceased excessive excretion and began to partially retract the cephalopodium. While this indicated a morphological recovery trend, these snails still exhibited a looser tissue posture and lacked the firm, active substrate attachment seen in the Control group.

### 3.3. Histological Alterations in Cephalopodium Tissue

**Figure 2:**
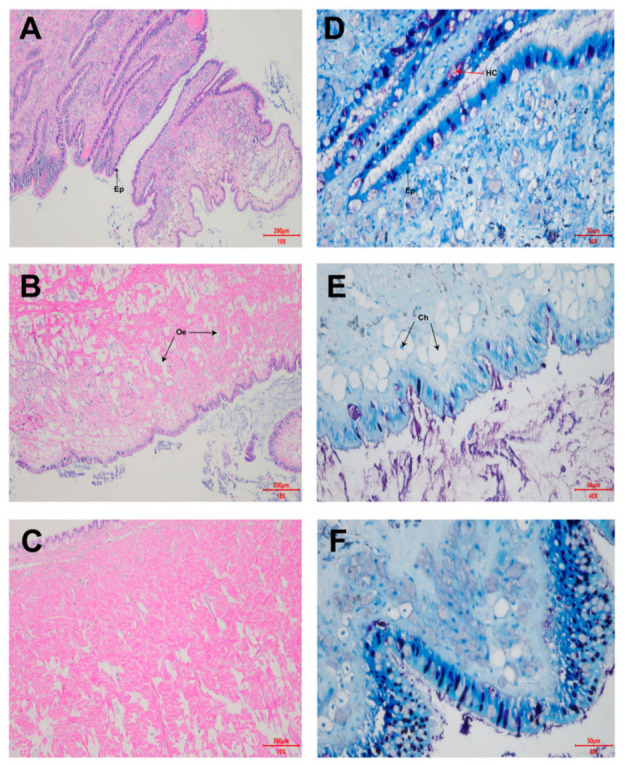
Representative histopathological alterations in the cephalopodium of *B. purificata* following exposure to *P. pulchellum* extract. **Left** Panel (H&E Staining at 100× magnification): (**A**) Control group showing intact tissue and a continuous epithelium (ep). (**B**) Treated group showing acute structural damage and severe interstitial oedema (oe). (**C**) Recovery group showing partial structural restoration with lingering muscle fibre separation. **Right** Panel (Nissl Staining at 400× magnification): (**D**) Control group displaying a well-defined epithelium (ep) containing normal, deeply stained healthy cells/Nissl bodies (hc). (**E**) Treated group exhibiting severe cellular stress and widespread chromatolysis. (**F**) Recovery group demonstrating intense basophilic staining, indicating incomplete cellular recovery.

To evaluate the tissue-level effects of *P. pulchellum* extract and the subsequent partial structural restoration of *B. purificata*, representative histological micrographs of the cephalopodium were assessed using Hematoxylin and Eosin (H&E) and Nissl staining across multiple magnifications (Figure 2). In the Control group, the cephalopodium exhibited a healthy, organized architecture. Medium-magnification (100×) structural assessment revealed a continuous, intact epithelial layer supported by dense sub-epithelial connective tissue and compactly arranged mucous glands (Figure 2A). High-magnification (400×) Nissl staining further confirmed normal cellular status, with distinct, deeply stained nuclei and well-defined cell boundaries throughout the tissue (Figure 2D).

Following exposure to the *P. pulchellum* extract, severe acute histopathological alterations were visually evident. Structurally, the tissue exhibited acute interstitial oedema, characterized by extensive vacuolation and the separation of muscle and connective tissue fibres (Figure 2B). Crucially, neurons and sub-epithelial cells displayed clear signs of chromatolysis, characterized by the dissolution of Nissl bodies and a loss of cytoplasmic staining intensity (Figure 2E), alongside the accumulation of irregular necrotic debris within the widened interstitial spaces.

In the Recovery group, the tissue architecture showed only partial structural restoration. Interstitial oedema had begun to subside, and the muscle fibres showed early stages of reorganization, though they still lacked the full compact density of the control morphology (Figure 2C). Furthermore, Nissl staining demonstrated intense hyperchromasia (darker staining) and nuclear condensation within the re-folded epithelial layer (Figure 2F), indicating that full cellular recovery and homeostasis had not yet been achieved within the 2-h timeframe.

### 3.4. Ultrastructural Changes (TEM)

**Figure 3:**
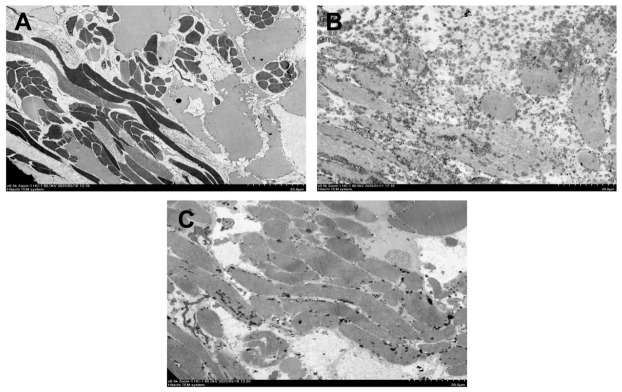
Transmission electron micrographs (TEMs) of the pedal musculature of *B. purificata* showing ultrastructural changes following exposure to *P. pulchellum* extract (Magnification: 500×). (**A**) Control group. (**B**) Treated group. (**C**) Recovery group. Scale bar = 20.0 µm.

Representative transmission electron microscopy (500×) micrographs revealed distinct morphological differences in the pedal musculature across the experimental groups. The Control group (Figure 3A) exhibited a compact and organized architecture, characterized by dense bundles of smooth muscle fibres separated by clearly defined, translucent interstitial spaces. Electron-dense granules within the sarcoplasm were unobtrusive and integrated within the dense muscle matrix.

In the Treated group (Figure 3B), the tissue displayed a marked loss of structural cohesiveness. The distinct boundaries between muscle bundles were obscured, and the interstitial spaces were occluded by a granular sarcoplasmic matrix, creating a loosened tissue appearance. This group was further distinguished by a high density of electron-dense granules that were unevenly dispersed throughout the sarcoplasm.

In the Recovery group (Figure 3C), the tissue architecture showed signs of partial structural reorganization. Although muscle fibre density remained lower than in the control, the bundles regained distinct boundaries, and clear interstitial spaces began to re-emerge. The electron-dense granules in this group were no longer widely dispersed but appeared in aggregated clusters.

### 3.5. Transcriptomic Analysis of B. purificata

#### 3.5.1. RNA-Seq Profiling of Control, Treated and Recovery Cephalopodium Tissue

RNA sequencing was performed on nine samples from Control (C), Treated (T), and Recovery (R) groups. Sequencing yielded 45–53 million reads per sample with high quality scores (Q30: 96–97%) and genome alignment rates ranged from 60 to 72% (Table 1). To characterize the gene functions, 20,105 unigenes were annotated using seven functional databases. Specifically, unigenes were annotated across the databases as follows: COG (3964; 19.7%), GO (8737; 43.4%), KEGG (7890; 39.2%), KOG (10,215; 50.8%), NR (19,593; 97.4%), Pfam (14,430; 71.8%), Swiss-Prot (12,482; 62.1%), and TrEMBL (19,333; 96.2%).

**Figure 4:**
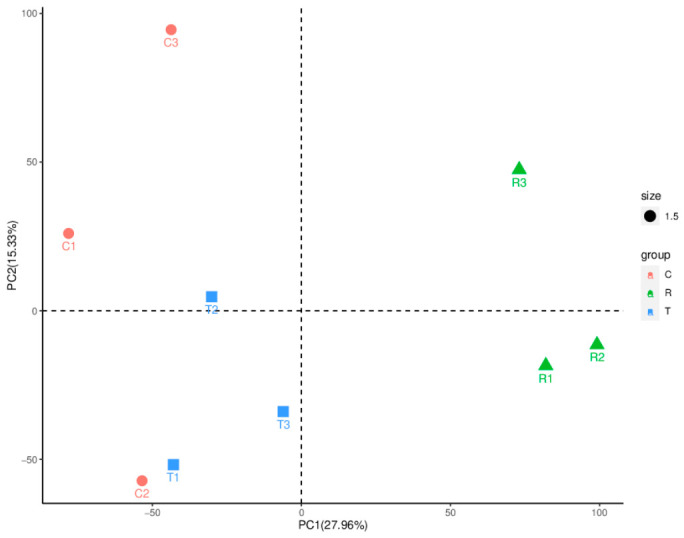
Principal Component Analysis (PCA) of *B. purificata* transcriptomes. PCA plot showing the distribution of samples from Control (C), Treated (T), and Recovery (R) groups based on global gene expression profiles. Each point represents a biological replicate (n = 3 per group).

To assess the global transcriptomic variability between groups, Principal Component Analysis (PCA) was performed (PC1: 27.96%, PC2: 27.33%). The PCA plot (Figure 4) revealed clear clustering of biological replicates within each group and distinct separation between the Control, Treated, and Recovery groups, indicating significant transcriptomic changes induced by the extract treatment.

#### 3.5.2. Differential Expression and Functional Enrichment Analysis

To identify genes associated with the response to *P. pulchellum* extract and the subsequent recovery process, differential expression analysis was performed for three pairwise comparisons: the Stress Response (Treated vs. Control; T vs. C), Recovery Process (Recovery vs. Treatment; R vs. T), and Residual Effects (Recovery vs. Control; R vs. C).

**Figure 5:**
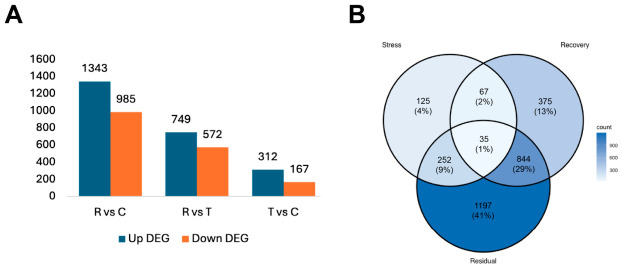
Transcriptomic overview of the stress and recovery response in *B. purificata*. (**A**) Statistics of differentially expressed genes (DEGs) across three comparisons: Residual Effects (R vs. C), Recovery Process (R vs. T), and Stress Response (T vs. C). Blue bars represent upregulated genes and orange bars represent downregulated genes. (**B**) Venn diagram illustrating the overlap of DEGs between the Stress Response (T vs. C), Recovery Process (R vs. T), and Residual Effects (R vs. C) groups.

The highest number of differentially expressed genes (DEGs) was observed in the Residual comparison, with 1343 upregulated and 985 downregulated genes (Figure 5A). The persistence of this high DEG count demonstrates that the global transcriptomic profile remained substantially altered compared to the baseline Control. In contrast, the Stress Response involved fewer DEGs (312 upregulated, 167 downregulated). A Venn diagram analysis revealed that 41% (1197 genes) of the total identified DEGs were unique to the Residual comparison, while only 4% (125 genes) were unique to the Stress phase (Figure 5B).

**Figure 6:**
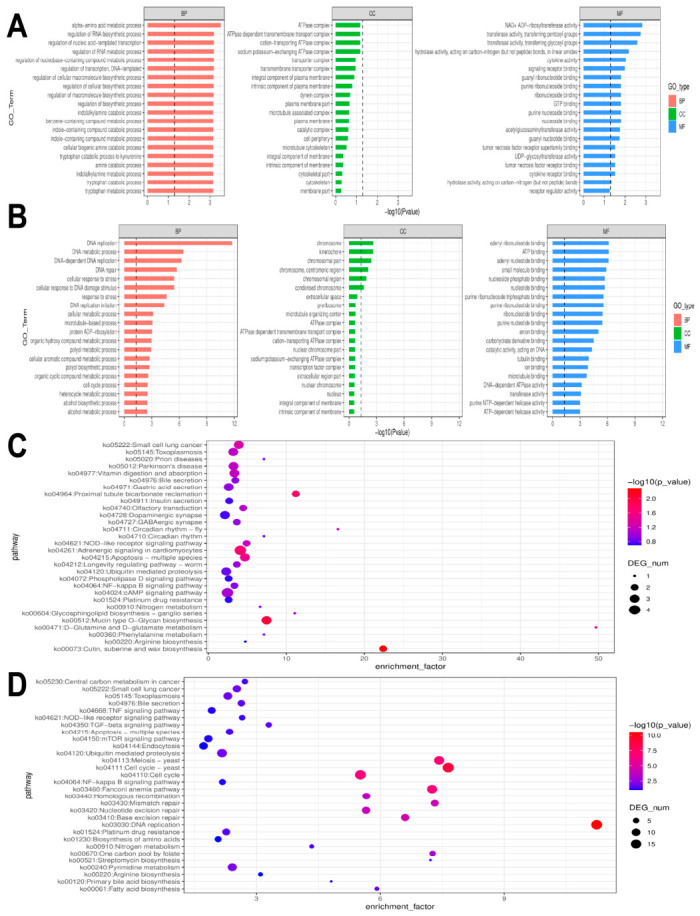
Gene Ontology (GO) and KEGG pathway enrichment analyses of Stress Response and Recovery Process differentially expressed genes (DEGs). (**A**) (GO) and (**C**) (KEGG) display the functional profile of the Stress Response (T vs. C). (**B**) (GO) and (**D**) (KEGG) show the functional profile of the Recovery Process (R vs. T). (Note: Bubble size corresponds to the number of DEGs, and colour represents the statistical significance, −log10(*p*-value).

Functional enrichment analysis was performed to distinguish the biological mechanisms driving the stress response versus those driving the recovery process (Figure 6). In the Stress Response analysis (T vs. C), Gene Ontology (GO) enrichment analysis identified a predominance of biological processes related to metabolic regulation, including alpha-amino acid metabolic process, regulation of RNA biosynthetic process, and tryptophan catabolic process (Figure 6A). KEGG pathway analysis (Figure 6C) revealed a coordinated physiological stress response. Notably, in response to the *P. pulchellum* extract, this included significant enrichment in conserved gene modules associated with apoptosis and homologous to vertebrate adrenergic signalling in cardiomyocyte. To further elucidate the molecular mechanisms driving these pathway enrichments, we analysed the differential expression of representative genes (Table 2). These pathways were driven by the upregulation of stress markers such as the cAMP-responsive element binding protein *CREB1* and the downregulation of the apoptosis inhibitor *BIRC7*.

The Recovery Process exhibited a functional profile distinct from the Stress Response, featuring transcriptomic signatures consistent with a DNA damage response. GO and KEGG analyses (Figure 6B,D) showed that while cell cycle and DNA replication pathways were enriched, the majority of genes within these pathways were downregulated (Table 2), indicating a cessation of cell proliferation. Specifically, the minichromosome maintenance (MCM) complex subunits *MCM2-6* were significantly downregulated. In contrast, the growth arrest and DNA damage-inducible gene *GADD45A* was significantly upregulated. Within the homologous recombination pathway, *RAD54B* was uniquely upregulated, whereas other repair factors in this pathway were downregulated.

#### 3.5.3. RT-qPCR Validation of Differentially Expressed Genes 

**Figure 7:**
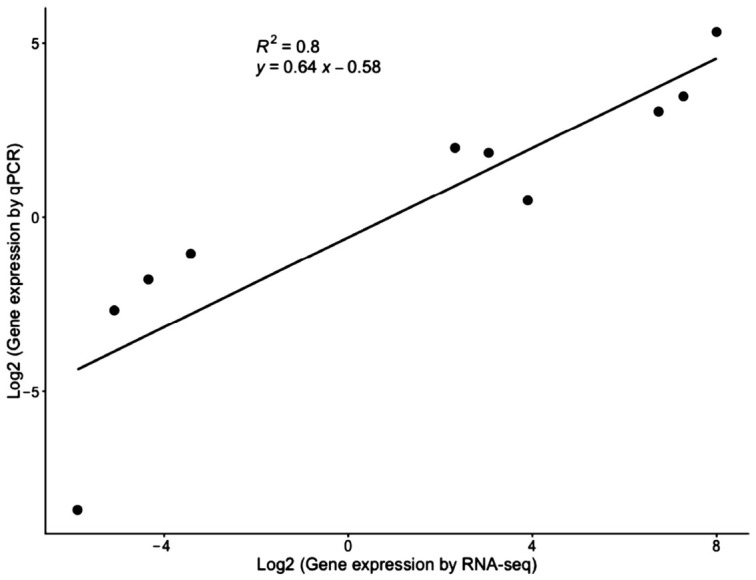
Correlation of gene expression fold changes between RNA-seq and RT-qPCR.

To verify the accuracy and reliability of the RNA-seq results, 7 representative expressed genes (*TDO*, *TRIM33*, *S100A16*, *SLC4A8*, *NEURL4*, *SSPO*, *LPR4*) were selected for RT-qPCR analysis. Expression levels were normalized against the reference gene (*β-actin*) using the 2^−ΔΔCt^ method. The specific primer sequences and complete gene identities are detailed in Supplementary Table S1. As shown in Figure 7, plotting the log_2_ fold changes of these genes yielded 10 comparison data points that were highly consistent with the high-throughput sequencing data. The strong positive correlation (R^2^ = 0.80) confirms the technical validity of the transcriptome sequencing platform used for subsequent downstream analysis.

## 4. Discussion

### 4.1. Behavioural, Physiological, and Molecular Mechanisms of Toxicity

The initial response of gastropods to chemical or exogenous stressors typically involves behavioural modifications aimed at minimizing toxicant uptake. In the present study, *B. purificata* exposed to *P. pulchellum* extract exhibited marked distress behaviours in the Treated group, specifically excessive mucus secretion and the active excretion and accumulation of faecal matter and soil. It is important to note that while the 80 mg/L concentration utilized in this study ensures a rapid and robust transcriptomic stress response, commercial production typically employs much lower dosages. In actual aquaculture practice, the extract is primarily used to induce this behavioral hyperextension and righting-reflex loss, with the bulk expulsion of mud subsequently facilitated by physical water exchanges.

Mucus hypersecretion functions as a physical protective barrier to reduce the diffusion of chemical irritants across the epithelial surface [23,24]. However, the subsequent progression to a state where the body remained fully expanded and unable to retract indicates an acute neuromuscular disruption. This loss of retraction capability is consistent with findings by Yap et al. [25], who reported that snails exposed to metabolic toxicants lost the ability to withdraw into their shells. Crucially, while Yap et al. described this as a passive inert state, the snails in our study maintained a sustained full extension accompanied by active depuration. This distinction is vital: rather than the lethargic collapse typical of general metabolic toxicity, our results suggest a profound state of neuromuscular dysfunction and severe metabolic stress.

This hypothesis is further supported by the accumulation of faecal matter. Typically, physiological stress impairs gut function; for instance, Gillis et al. [26] reported that *Daphnia magna* showed reduced clearance of gut sediments under metal stress. In contrast, the increased egestion rates and sustained hyperactivity state observed in our study suggest a severe disruption of normal neuro-intestinal regulation, overriding standard stress-induced behavioural inhibition.

Our chemical profiling of the *P. pulchellum* extract (detailed in Supplementary Table S2) characterized its primary bioactive constituents, identifying compounds such as rutin, betaine, kaempferol-3-O-rutinoside, kaempferol 3-glucorhamnoside, and morin. Notably, while exogenous indole alkaloids or tryptamine derivatives were not detected, the transcriptomic data reveals expression patterns consistent with a massive endogenous neuroendocrine disruption. The GO enrichment analysis for Stress Response (Figure 5B) revealed a significant upregulation of the tryptophan catabolic process and tryptophan metabolic process. As tryptophan is the endogenous precursor to the snail’s native serotonin [27,28], a key neurotransmitter regulating gastropod gut motility and pedal locomotion [29,30,31]. The enrichment of these pathways indicates a severe metabolic stress response to the extract’s primary toxicants. Previous studies on *B. purificata* have demonstrated that environmental stressors induce profound alterations in the biosynthesis and metabolism of amino acids and lipids within the foot muscle [32]. In the present study, the acute extract exposure likely overwhelmed the metabolic capacity, triggering a dysregulated neuroendocrine response that manifested physically as the sustained depuration reflex and inability to retract.

Beyond these behavioural modifications, the extract induced significant internal tissue damage that creates a structural basis for the observed neuromuscular failure. Histological examination of the pedal sole revealed acute interstitial oedema. Notably, neurons and sub-epithelial cells displayed clear signs of chromatolysis (Figure 2E). Chromatolysis is a hallmark of neuronal injury and axon regeneration failure [33,34]. This cellular stress response is often triggered by prolonged hyperexcitation or metabolic exhaustion, mechanisms known to disrupt ionic gradients and deplete cellular energy reserves [35]. This specific finding identifies the nervous system as a direct primary target of the extract, physically supporting the neuro-behavioural dysfunction hypothesis proposed above.

To further characterize this damage, TEM revealed the treated tissue displayed a marked loss of structural cohesiveness (Figure 3B). This physical uncoupling of muscle fibres likely compromised contractile force, explaining the behavioural inability to retract. However, the partial restoration of bundle boundaries in the Recovery group suggests that this structural disruption may be reversible rather than necrotic. Furthermore, the Treated group exhibited a chaotic dispersion of electron-dense granules throughout the sarcoplasm. These granules likely reflect the rapid mobilization of putative energy stores to meet the high metabolic energy demand of the sustained neuromuscular hyperextension [36], or stress-induced protein aggregates. While further histochemical validation would be required to definitively confirm their biochemical identity as glycogen, the sustained neuromuscular locking suggests that metabolic fuel mobilization is only part of the story.

The transcriptomic data provides the molecular context for this rigidity, revealing that a significant enrichment of conserved gene modules homologous to the vertebrate adrenergic signalling in the cardiomyocyte pathway was accompanied by a substantial upregulation of the 16 kDa calcium-binding protein-like gene. Although named for cardiac tissue, this pathway represents a conserved mechanism of calcium-dependent muscle contraction. Adrenergic signalling is known to mediate massive calcium influx [37,38]. The upregulation of this calcium-binding protein suggests a compensatory mechanism to buffer cytosolic calcium overload. Consequently, while the electron-dense granules may primarily represent mobilized energy stores, the transcriptomic data suggests that a potential intracellular calcium disorder likely provides the molecular basis for the neuromuscular locking and sustained body extension described above.

Finally, the extensive tissue damage is mechanistically consistent with the significant downregulation of baculoviral IAP repeat-containing protein 7-like (*BIRC7*), a member of the Inhibitor of Apoptosis Protein (IAP) family. Under normal conditions, IAPs protect cells from stress-induced death by inhibiting caspases [39,40,41]. This depletion of the protective IAP factor compromised the tissue’s defence mechanisms. This allowed the extract to cause structural damage and muscle uncoupling, bringing the tissue to the absolute edge of cell death without causing the permanent, irreversible damage that would prevent recovery. However, this proximity to cellular collapse explains the persistent transcriptomic alterations observed in the present study. The massive DEG count in the Residual (Recovery vs. Control) comparison demonstrates that while the organisms exhibited partial morphological and behavioural restoration within the 2-h window, profound molecular repair mechanisms remained highly active, and full physiological homeostasis had not yet been achieved. Furthermore, because ultrastructural and transcriptomic alterations frequently exhibit a temporal lag relative to rapid behavioural shifts, this 2-h observation window may only capture the initial phases of biological recovery.

### 4.2. Molecular Mechanisms of Genomic Stress and Delayed DNA Repair

The acute impact of the *P. pulchellum* extract extends beyond cellular structural damage, eliciting transcriptomic signatures consistent with a DNA damage response. As previously established in our chemical profiling, the extract contains several potent flavonoids, including rutin, kaempferol-3-O-rutinoside, and morin. Acute exposure to these bioactive phytochemicals can induce oxidative stress through the rapid generation of reactive oxygen species (ROS), which can subsequently lead to DNA strand breaks [42,43]. While direct genotoxicity assays (e.g., comet assay or ROS quantification) are required to definitively confirm physical DNA fragmentation, recent physiological evaluations of *B. purificata* confirm this specific vulnerability, demonstrating that acute environmental stress rapidly overwhelms the snail’s antioxidant defence system, resulting in unmitigated ROS accumulation [44].

During the Stress Response phase, this cellular insult appears to have overwhelmed cellular defences, effectively removing the biochemical brakes on cell death, as evidenced by the downregulation of survival factors (*BIRC7*) described in Section 4.1. However, the transcriptomic profile of the Recovery Process suggests a distinct, delayed molecular effort to restore genomic integrity. This priority is statistically underscored by the GO analysis, where the top seven most significantly enriched pathways were dominantly associated with DNA maintenance (e.g., DNA replication, DNA repair, DNA metabolic process) and stress adaptation (e.g., cellular response to DNA damage stimulus, cellular response to stress).

Within the cell cycle pathway, the *GADD45* gene emerged as a putative molecular marker of the Recovery Process, exhibiting robust upregulation in response to the extract-induced stress. GADD45 acts as a genomic stress sensor that is induced under oxidative and genotoxic stresses, resulting in regulation of DNA repair, cell cycle arrest and apoptosis [45,46,47]. Its upregulation suggests that the surviving cells initiated a coordinated checkpoint activation following the removal of the extract. This mirrors findings in other molluscan models; for instance, comparable overexpression of *GADD45* served as a critical signal for DNA repair following toxicant exposure [48,49,50].

Furthermore, the enrichment of the homologous recombination pathway suggests a transcriptomic response to potential double-strand breaks, which are frequently induced by ROS-mediated oxidative stress [51]. The transcriptomic data shows the differential expression of key HR components, including the significant upregulation of the repair scaffold gene *RAD54B* [52,53], alongside the concurrent downregulation of the core recombinase RAD51. Because HR is a highly complex process, inferring specific repair kinetics, such as a delayed repair mechanism, from these opposing expression patterns remains speculative based solely on transcriptomic data. Future studies incorporating a broader suite of HR pathway genes and direct physical assays of DNA repair are required to fully elucidate these dynamics.

Functionally, GADD45 proteins arrest the cell cycle at the G2/M checkpoint, serving as a protective mechanism that delays cell division to allow DNA repair pathways to excise and repair damaged segments before replication proceeds [45,47,54,55]. These transcriptomic signatures of an active process likely explain the histological observation in the Recovery group (Figure 3C), where tissue architecture began to reorganize but muscle fibre density remained lower than in controls. This massive transcriptomic alteration, particularly the sustained activation of these repair pathways, demonstrates that while the organisms exhibited partial morphological and behavioural restoration within the 2-h window, profound molecular repair mechanisms remained highly active, and full physiological homeostasis had not yet been achieved.

This mechanism is further supported by the profound downregulation of the MCM complex genes (*MCM2-6*; Table 2). The MCM complex forms the core of the replicative helicase, essential for the initiation of DNA replication forks [56,57,58]. The suppression of these genes, along with Replication Protein A (*RPA*), which plays an important role in the DDR [58], signifies a negative regulation or systemic blockage of DNA synthesis. While this suppression could signify a controlled physiological response to delay the replication machinery and prevent the propagation of damaged DNA, it must be noted that this global downregulation may also reflect general metabolic suppression, reduced cellular proliferation, or tissue composition changes resulting from the severe acute phytochemical stress.

In summary, the molecular data paints a picture of resilience. While the KEGG analysis highlighted broad enrichment in the cell cycle and DNA replication pathways, the specific enrichment of DNA repair and response to DNA damage stimulus pathways in GO terms, coupled with the activation of homologous recombination and *GADD45*-mediated arrest, suggests that *B. purificata* mounts a coordinated transcriptomic stress-response system to halt proliferation and prioritize genomic integrity following phytochemical-induced cellular stress. However, because this study assessed only a 2-h recovery window, conclusions regarding the ultimate reversibility of the tissue damage and the extract’s viability for industrial application remain premature. Future studies incorporating extended recovery periods, long-term survival tracking, and comprehensive food-safety assessments are imperative before establishing the extract’s overall safety for commercial depuration processes.

## 5. Conclusions

This study provides the first comprehensive molecular and physiological characterization of the acute stress response in *B. purificata* induced by *P. pulchellum* extract, which facilitates rapid industrial depuration. While highly effective at accelerating intestinal clearance, the extract triggers acute, systemic stress characterized by sustained neuromuscular dysfunction and structural tissue loosening. This behavioural shift is accompanied by profound endogenous neuroendocrine disruption, potential intracellular calcium dysregulation, and the rapid mobilization of putative energy stores. Importantly, while the extract induces significant cellular stress and transcriptomic signatures indicative of DNA damage, surviving tissues mount a robust, delayed molecular repair response, systemically suppressing the cell cycle to prioritize genomic repair. Ultimately, by decoding these transcriptomic mechanisms and demonstrating the partial short-term structural restoration of cellular damage, this study provides a strong biological foundation for highly efficient depuration protocols. However, as this study did not include direct physiological or biochemical indicator assays, future investigations incorporating extended recovery periods, physiological validation, and comprehensive food-safety assessments remain essential to fully validate the ultimate reversibility of this stress and its commercial safety for the snail food industry.

## Figures and Tables

**Table 1:** Summary statistics for the transcriptome sequencing data from each sample.

Sample	Total Reads	Total Base	Q30 (%)	Pair Reads	Total Alignment Ratio
C1	53,360,024	8,004,003,600	96.14	26,680,012	72.19%
C2	45,961,158	6,894,173,700	96.91	22,980,579	60.88%
C3	49,045,206	7,356,780,900	97.00	24,522,603	62.23%
R1	46,605,078	6,990,761,700	96.77	23,302,539	67.95%
R2	48,428,386	7,264,257,900	96.55	24,214,193	64.37%
R3	46,293,162	6,943,974,300	96.66	23,146,581	67.00%
T1	45,589,184	6,838,377,600	96.93	22,794,592	67.55%
T2	53,212,058	7,981,808,700	97.05	26,606,029	67.11%
T3	45,897,766	6,884,664,900	96.60	22,948,883	64.93%

**Table 2:** Differential expression of representative genes from significantly enriched KEGG pathways in *B. purificata*.

KEGG Pathway	Gene Name	Log_2_FC
Stress Response (T vs. C)		
Adrenergic signalling in cardiomyocytes	16 kDa calcium-binding protein-like (*S100A16*)	3.05
	Cyclic AMP-responsive element-binding protein 1-like isoform X2 (*CREB1*)	1.56
	Adenylate cyclase type 3-like (*AC3*)	−2.54
Apoptosis	Baculoviral IAP repeat-containing protein 7-like (*BIRC7*)	−1.44
Recovery Process (R vs. T)		
Cell cycle	Cell division cycle protein 20 homolog (*CDC20*)	2.00
	Growth arrest and DNA damage-inducible protein GADD45 alpha-like isoform X2 (*GADD45A*)	1.28
	Minichromosome maintenance complex subunits (*MCM2-6*)	−2.39 to −1.98
DNA replication	Minichromosome maintenance complex subunits (*MCM2-6*)	−2.08 to −1.98
	Replication protein A 32 kDa subunit-B-like isoform X2 (*RPA2*)	−1.63
Homologous recombination	DNA repair and recombination protein RAD54B-like (*RAD54B*)	2.23
	DNA repair protein RAD51 homolog 1 (*RAD51*)	−1.06

## Data Availability

The raw transcriptomic sequencing data generated in this study have been deposited in the National Center for Biotechnology Information (NCBI) database and are publicly accessible under BioProject accession number PRJNA1442220.

## Supplementary Materials

The following supporting information can be downloaded at: https://www.mdpi.com/article/10.3390/ani16101490/s1, Figure S1: Total ion chromatograms (TIC) of the *Phyllodium pulchellum* botanical extract obtained via UHPLC-MS/MS analysis in (A) positive and (B) negative ion modes; Table S1: RT-qPCR primer list; Table S2: Top 20 most abundant phytochemical constituents of the *Phyllodium pulchellum* extract identified via UHPLC-MS/MS.

## References

[1] Liu Y., Zhang W., Wang Y. (1979). Economic Fauna of China (Freshwater Mollusca).

[2] Jin W., Cao X.J., Ma X.Y., Lv G.H., Xu G.C., Xu P., Sun B., Xu D.-P., Wen H.-B. (2022). Chromosome-level genome assembly of the freshwater snail *Bellamya purificata* (Caenogastropoda). Zool. Res..

[3] Wang C., Zhou H. (2025). Development status and countermeasures of the Liuzhou Luosifen industry. China Food Ind..

[4] Huang Y., Zhang J., Xu D., Ren X., Yang W., Lu K., Zhu J. (2024). Metabolomics revealed that toxic cyanobacteria stress reduced the flavor quality of *Bellamya aeruginosa*. Front. Sustain. Food Syst..

[5] Ma T., Zhou K., Zhu C., Liu J., Wang Z.J. (2009). Biomarker responses of *Bellamya aeruginosa* to chronic stress of cadmium-contaminated sediments. Acta Sci. Circumstantiae.

[6] Ozawa K., Yokoyama A., Ishikawa K., Kumagai M., Watanabe M.F., Park H.D. (2003). Accumulation and depuration of microcystin produced by the cyanobacterium Microcystis in a freshwater snail. Limnology.

[7] Zamora L.N., Burritt D.J., Ragg N.L., Casanovas P., Delorme N.J. (2025). Gaping restriction as a mechanism to mitigate live transport and re-immersion physiological stress in mussels. Aquaculture.

[8] Feng J., Chai L., Liu B., Chen X. (2022). Preparation and quality analysis of total alkaloids extract from *Desmodium pulchellum*. Guangxi J. Tradit. Chin. Med..

[9] Gao S., Yu L., Cao K., Ouyang S. (2022). Protective effect of total alkaloids from *Desmodium pulchellum* against acute liver failure in rats by inhibiting NF-κB pathway. Drugs Clin..

[10] Luo F., Wen Y., Wang Z., Yu X., Chen K., Liao Z., Qin S. (2023). A Chinese Herbal Preparation for Stimulating The Shell-Out Activity of Freshwater Snails and Its Application.

[11] Wang L., Song X., Song L. (2018). The oyster immunity. Dev. Comp. Immunol..

[12] Song L., Wang L., Zhang H., Wang M. (2015). The immune system and its modulation mechanism in scallop. Fish Shellfish Immunol..

[13] Liu Z., Li M., Yi Q., Wang L., Song L. (2018). The neuroendocrine-immune regulation in response to environmental stress in marine bivalves. Front. Physiol..

[14] Huang Q., Li Q., Chen H., Lin B., Chen D. (2019). Neuroendocrine immune-regulatory of a neuropeptide ChGnRH from the Hongkong oyster, *Crassostrea Hongkongensis*. Fish Shellfish Immunol..

[15] Huang W., Xu F., Qu T., Zhang R., Li L., Que H., Zhang G. (2015). Identification of thyroid hormones and functional characterization of thyroid hormone receptor in the pacific oyster Crassostrea gigas provide insight into evolution of the thyroid hormone system. PLoS ONE.

[16] Li W., Zhang L., Zhang M., Li R., Li Y., Guo Z., Bao Z. (2020). Cloning and expression analysis of gonadotropin-releasing hormone gene in bay scallop. J. Ocean Univ. China (Nat. Sci. Ed.).

[17] Smit A.B., Vreugdenhil E., Ebberink R.H.M., Geraerts W.P.M., Klootwijk J., Joosse J. (1988). Growth-controlling molluscan neurons produce the precursor of an insulin-related peptide. Nature.

[18] Fan Y.C., Yue S.J., Guo Z.L., Xin L.T., Wang C.Y., Zhao D.L., Guan H.-S., Wang C.-Y. (2018). Phytochemical composition, hepatoprotective, and antioxidant activities of *Phyllodium pulchellum* (L.) Desv. Molecules.

[19] Chen F.L., Zhang H.S., Yang J., Chai L., Zhong M., Liu B., Yuan J., Jiang Z.-H., Zhu G.-Y. (2021). Phytochemical and chemotaxonomic studies on *Phyllodium pulchellum* (Leguminosae). Biochem. Syst. Ecol..

[20] Tarazona S., García-Alcalde F., Dopazo J., Ferrer A., Conesa A. (2011). Differential expression in RNA-seq: A matter of depth. Genome Res..

[21] Marioni J.C., Mason C.E., Mane S.M., Stephens M., Gilad Y. (2008). RNA-seq: An assessment of technical reproducibility and comparison with gene expression arrays. Genome Res..

[22] Zhang S., Chen Y., Wang Y., Zhang P., Chen G., Zhou Y. (2020). Insights into translatomics in the nervous system. Front. Genet..

[23] Rashad M., Sampò S., Cataldi A., Zara S. (2025). From Nature to Nurture: The Science and Applications of Snail Slime in Health and Beauty. J. Cosmet. Dermatol..

[24] Sullivan J.T., Cheng T.C. (1975). Heavy metal toxicity to *Biomphalaria glabrata* (Mollusca: Pulmonata). Ann. N. Y. Acad. Sci..

[25] Yap C.K., Pang B.H., Cheng W.H., Kumar K., Avtar R., Okamura H., Horie Y., Sharifinia M., Keshavarzifard M., Ong M.C. (2023). Heavy metal exposures on freshwater snail *Pomacea insularum*: Understanding its biomonitoring potentials. Appl. Sci..

[26] Gillis P.L., Chow-Fraser P., Ranville J.F., Ross P.E., Wood C.M. (2005). Daphnia need to be gut-cleared too: The effect of exposure to and ingestion of metal-contaminated sediment on the gut-clearance patterns of *D. magna*. Aquat. Toxicol..

[27] Xie X., Ding D., Bai D., Zhu Y., Sun W., Sun Y., Zhang D. (2022). Melatonin biosynthesis pathways in nature and its production in engineered microorganisms. Synth. Syst. Biotechnol..

[28] Wichansawakun S., Chupisanyarote K., Wongpipathpong W., Kaur G., Buttar H.S. (2022). Antioxidant diets and functional foods attenuate dementia and cognition in elderly subjects. Functional Foods and Nutraceuticals in Metabolic and Non-Communicable Diseases.

[29] McKenzie J.D., Caunce M., Hetherington M.S., Winlow W. (1998). Serotonergic innervation of the foot of the pond snail *Lymnaea stagnalis* (L.). J. Neurocytol..

[30] Fong P.P., Molnar N. (2013). Antidepressants cause foot detachment from substrate in five species of marine snail. Mar. Environ. Res..

[31] Tecott L.H. (2007). Serotonin and the orchestration of energy balance. Cell Metab..

[32] Lou Y., Jia R., Li B., Zhou L., Zhu J., Hou Y. (2024). Effects of Different Stocking Densities on Snail *Bellamya purificata* Foot Muscle Nutritional Quality and Metabolic Function. Animals.

[33] Moon L.D.F. (2018). Chromatolysis: Do injured axons regenerate poorly when ribonucleases attack rough endoplasmic reticulum, ribosomes and RNA?. Dev. Neurobiol..

[34] Johnson I.P., Sears T.A. (2013). Target-dependence of sensory neurons: An ultrastructural comparison of axotomised dorsal root ganglion neurons with allowed or denied reinnervation of peripheral targets. Neuroscience.

[35] Massieu L., Garcia O. (1998). The role of excitotoxicity and metabolic failure in the pathogenesis of neurological disorders. Neurobiology.

[36] Alonso Á. (2023). Previous stress causes a contrasting response to cadmium toxicity in the aquatic snail *Potamopyrgus antipodarum*: Lethal and behavioural endpoints. Environ. Sci. Pollut. Res..

[37] Papa A., Kushner J., Marx S.O. (2022). Adrenergic regulation of calcium channels in the heart. Annu. Rev. Physiol..

[38] Zhou P., Zhao Y.T., Guo Y.B., Xu S.M., Bai S.H., Lakatta E.G., Cheng H., Hao X.-M., Wang S.-Q. (2009). β-Adrenergic signaling accelerates and synchronizes cardiac ryanodine receptor response to a single L-type Ca^2+^ channel. Proc. Natl. Acad. Sci. USA.

[39] Silke J., Meier P. (2013). Inhibitor of apoptosis (IAP) proteins–modulators of cell death and inflammation. Cold Spring Harb. Perspect. Biol..

[40] Berthelet J., Dubrez L. (2013). Regulation of apoptosis by inhibitors of apoptosis (IAPs). Cells.

[41] Kocab A.J., Duckett C.S. (2016). Inhibitor of apoptosis proteins as intracellular signaling intermediates. FEBS J..

[42] Chobot V., Hadacek F. (2011). Exploration of pro-oxidant and antioxidant activities of the flavonoid myricetin. Redox Rep..

[43] Procházková D., Boušová I., Wilhelmová N. (2011). Antioxidant and prooxidant properties of flavonoids. Fitoterapia.

[44] Sui C., Liu M., Chuan S., Wang B., Zhang T., Zhang W., Huang R., Qiu Z., Wang Y., Zhao W. (2024). Responses of survival, antioxidant system and intestinal microbiota of native snail *Bellamya purificata* to the invasive snail *Pomacea canaliculata*. Sci. Rep..

[45] Liebermann D.A., Hoffman B. (2008). Gadd45 in stress signaling. J. Mol. Signal..

[46] Tamura R.E., de Vasconcellos J.F., Sarkar D., Libermann T.A., Fisher P.B., Zerbini L.F. (2012). GADD45 proteins: Central players in tumorigenesis. Curr. Mol. Med..

[47] Moskalev A., Plyusnina E., Shaposhnikov M., Shilova L., Kazachenok A., Zhavoronkov A. (2012). The role of D-GADD45 in oxidative, thermal and genotoxic stress resistance. Cell Cycle.

[48] Slobodskova V.V., Solodova E.E., Slinko E.N., Chelomin V.P. (2010). Evaluation of the genotoxicity of cadmium in gill cells of the clam *Corbicula japonica* using the comet assay. Russ. J. Mar. Biol..

[49] Slobodskova V.V., Zhukovskaya A.F., Chelomin V.P. (2012). DNA damage in the gill cells of the marine scallop *Mizuhopecten yessoensis* during anoxic stress and aerobic recovery. Ocean Sci. J..

[50] de Boissel P.G.J., Fournier M., Rodriguez-Lecompte J.C., McKenna P., Kibenge F., Siah A. (2017). Functional and molecular responses of the blue mussel Mytilus edulis’ hemocytes exposed to cadmium—An in vitro model and transcriptomic approach. Fish Shellfish Immunol..

[51] Srinivas U.S., Tan B.W., Vellayappan B.A., Jeyasekharan A.D. (2019). ROS and the DNA damage response in cancer. Redox Biol..

[52] Liu P., Lin C., Liu L., Lu Z., Tu Z., Liu H. (2022). RAD54B mutations enhance the sensitivity of ovarian cancer cells to poly (ADP-ribose) polymerase (PARP) inhibitors. J. Biol. Chem..

[53] Yasuhara T., Suzuki T., Katsura M., Miyagawa K. (2014). Rad54B serves as a scaffold in the DNA damage response that limits checkpoint strength. Nat. Commun..

[54] Salerno D.M., Tront J.S., Hoffman B., Liebermann D.A. (2012). Gadd45a and Gadd45b modulate innate immune functions of granulocytes and macrophages by differential regulation of p38 and JNK signaling. J. Cell. Physiol..

[55] Maeda T., Hanna A.N., Sim A.B., Chua P.P., Chong M.T., Tron V.A. (2002). GADD45 regulates G2/M arrest, DNA repair, and cell death in keratinocytes following ultraviolet exposure. J. Investig. Dermatol..

[56] Forsburg S.L. (2004). Eukaryotic MCM proteins: Beyond replication initiation. Microbiol. Mol. Biol. Rev..

[57] You Z., Masai H. (2024). Assembly, Activation, and Helicase Actions of MCM2-7: Transition from Inactive MCM2-7 Double Hexamers to Active Replication Forks. Biology.

[58] Sampathkumar A., Zhong C., Tang Y., Fujita Y., Ito M., Shinohara A. (2024). Replication protein-A, RPA, plays a pivotal role in the maintenance of recombination checkpoint in yeast meiosis. Sci. Rep..

