# Supplementary material for: Transcriptomic and Ultrastructural Analyses Reveal the Mechanisms of Accelerated Depuration Induced by Phyllodium pulchellum Extract in the Freshwater Snail Bellamya purificata
Source: Animals (Basel). 2026 May 13;16(10):1490. doi: 10.3390/ani16101490 (PMC13203559; doi:10.3390/ani16101490)
Supplement: Supplementary file 1 [file animals-16-01490-s001.zip › animals-4284010-supplementary.pdf]

Supplementary Material

Supplementary Table S1. RT-qPCR primer list.

Gene Name	Forward Primer (5'–3')	Reverse Primer (5'–3')
β-actin (Reference)	GGCAGGTCTTCCAATCAC	TTCATCTTGCTCCTCACG
TDO	CAAGTGCCCTCATATGGTTG	TCGAAGGTCTCCTCGTTTCC
TRIM33	GTTGATGTCAAACGAACCAATG	TCTTCACAAACTCCAAGCACTG
S100A16	TCTATCTTCCTGGACCTGGACTCA	ACTCTCGAAACATTTTTTCTGCCT
SLC4A8	CGACTTCAAGGACGCAATACAC	CCAAAGAACGCAAAAAATACAC
NEURL4	AGCAGACCCAGAGATGGAAGAA	AACTGACCAGACAGTCACGACA
SSPO	TAGCAGGAACAAATGGGAGATA	TGAAAGCCAACCGACGTAAATA
LRP4	CTATTTTAGGGGAAAGAGGGGA	TGGTCAGAGACGATGTGGATGT

Supplementary Table S2. Top 20 most abundant phytochemical constituents of the *Phyllodium pulchellum* extract identified via UHPLC-MS/MS.

Compound Name	Molecular Formula	Retention Time (min)	Relative Abundance (%)
Rutin	C27H30O16	24.606	23.650
Betaine	C5H11NO2	1.533	14.246
Kaempferol-3-O-rutinoside	C27H30O15	25.405	12.291
Kaempferol 3-glucorhamnoside	C27H30O15	25.140	5.761
Morin	C15H10O7	24.614	5.239
Quercetin	C15H10O7	29.300	3.289
Hyperoside	C21H20O12	25.081	2.710
Abietic acid	C20H30O2	47.634	2.682
Kaempferol	C15H10O6	25.410	2.679
Isoquercitrin	C21H20O12	24.615	1.857

Trigonelline HCl	$C_7H_7NO_2$	1.581	1.855
Epigallocatechin	$C_{15}H_{14}O_7$	22.347	1.465
Artemetin	$C_{20}H_{20}O_8$	38.738	1.225
Guanine	$C_5H_5N_5O$	1.579	1.200
Lactose	$C_{12}H_{22}O_{11}$	1.576	1.109
Astragalin	$C_{21}H_{20}O_{11}$	25.409	1.030
(+)-Catechin hydrate	$C_{15}H_{14}O_6$	23.495	0.804
Sucrose	$C_{12}H_{22}O_{11}$	1.576	0.725
(-)-Gallocatechin	$C_{15}H_{14}O_7$	22.343	0.657
Caffeic acid	$C_9H_8O_4$	21.715	0.598

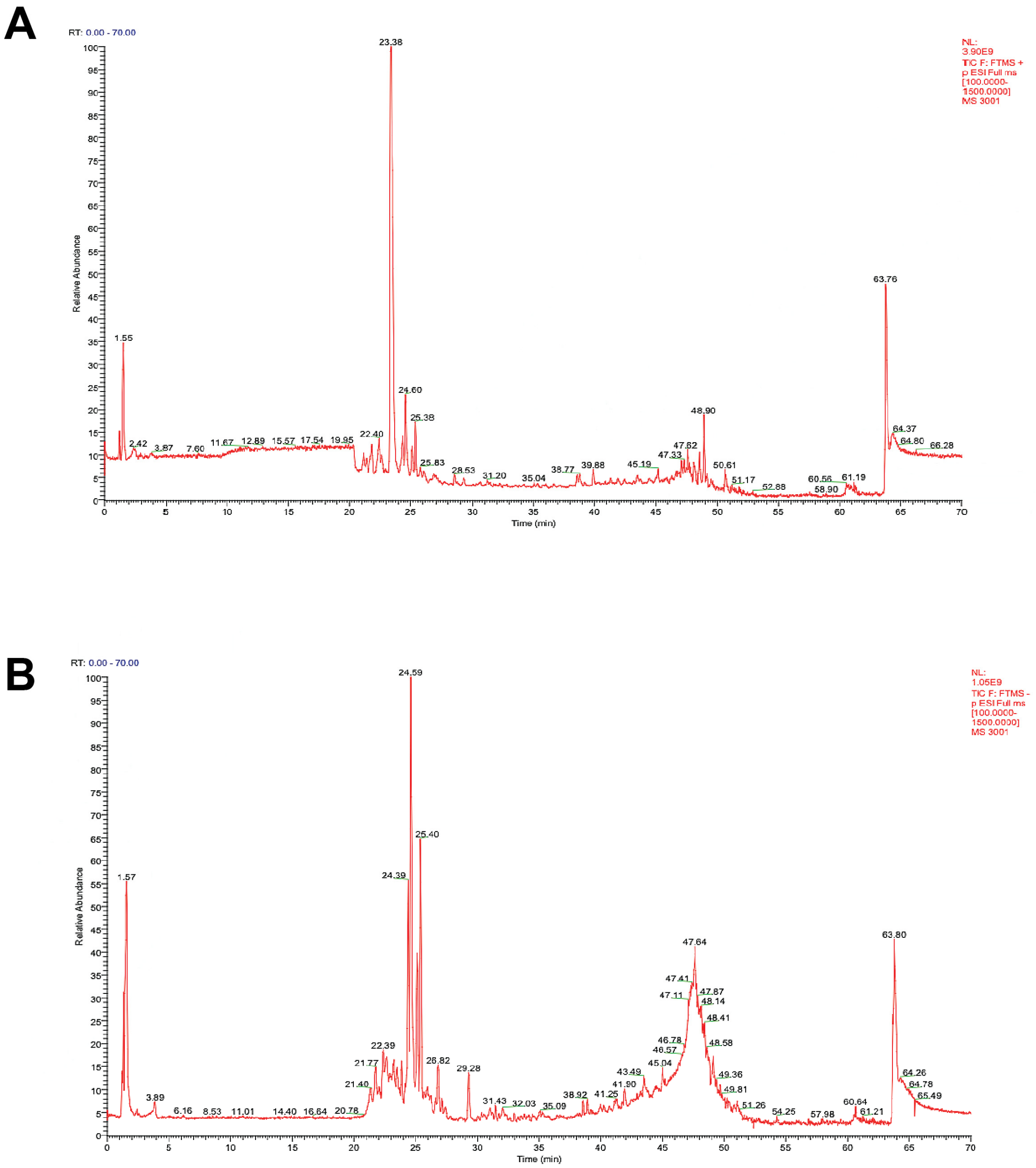

Supplementary Figure S1. Total ion chromatograms (TIC) of the *Phyllodium pulchellum* botanical extract obtained via UHPLC-MS/MS analysis in (A) positive and (B) negative ion modes.
